# Duration of Untreated Prodromal Psychosis and Cognitive Impairments

**DOI:** 10.1001/jamanetworkopen.2023.53426

**Published:** 2024-01-26

**Authors:** TianHong Zhang, HuiRu Cui, YanYan Wei, XiaoChen Tang, LiHua Xu, YeGang Hu, YingYing Tang, HaiChun Liu, ZiXuan Wang, Tao Chen, ChunBo Li, JiJun Wang

**Affiliations:** 1 Shanghai Mental Health Center, Shanghai Jiaotong University School of Medicine, Shanghai Engineering Research Center of Intelligent Psychological Evaluation and Intervention, Shanghai Key Laboratory of Psychotic Disorders, Shanghai, PR China; 2 Department of Automation, Shanghai Jiao Tong University, Shanghai, China; 3 Shanghai Xinlianxin Psychological Counseling Co Ltd, Shanghai, PR China; 4 Big Data Research Lab, University of Waterloo, Waterloo, Ontario, Canada; 5 Labor and Worklife Program, Harvard University, Cambridge, Massachusetts; 6 Center for Excellence in Brain Science and Intelligence Technology, Chinese Academy of Science, Shanghai, PR China; 7 Institute of Psychology and Behavioral Science, Shanghai Jiao Tong University, Shanghai, PR China

## Abstract

**Importance:**

The possible association between the duration of untreated prodromal symptoms (DUPrS) and cognitive functioning in individuals at clinical high risk (CHR) for psychosis remains underexplored.

**Objective:**

To investigate the intricate interplay between DUPrS, cognitive performance, and conversion outcomes, shedding light on the potential role of DUPrS in shaping cognitive trajectories and psychosis risk in individuals at CHR for psychosis.

**Design, Setting, and Participants:**

This cohort study of individuals at CHR for psychosis was conducted at the Shanghai Mental Health Center in China from January 10, 2016, to December 29, 2021. Participants at CHR for psychosis typically exhibit attenuated positive symptoms; they were identified according to the Structured Interview for Prodromal Syndromes, underwent baseline neuropsychological assessments, and were evaluated at a 3-year clinical follow-up. Data were analyzed from August 25, 2021, to May 10, 2023.

**Exposure:**

Duration of untreated prodromal symptoms and cognitive impairments in individuals at CHR for psychosis.

**Main Outcomes and Measures:**

The primary study outcome was conversion to psychosis. The DUPrS was categorized into 3 groups based on percentiles (33rd percentile for short [≤3 months], 34th-66th percentile for median [4-9 months], and 67th-100th percentile for long [≥10 months]). The DUPrS, cognitive variables, and the risk of conversion to psychosis were explored through quantile regression and Cox proportional hazards regression analyses.

**Results:**

This study included 506 individuals (median age, 19 [IQR, 16-21] years; 53.6% [n = 271] women). The mean (SD) DUPrS was 7.8 (6.857) months, and the median (IQR) was 6 (3-11) months. The short and median DUPrS groups displayed poorer cognitive performance than the long DUPrS group in the Brief Visuospatial Memory Test–Revised (BVMT-R) (Kruskal-Wallis χ^2^ = 8.801; *P* = .01) and Category Fluency Test (CFT) (Kruskal-Wallis χ^2^ = 6.670; *P* = .04). Quantile regression analysis revealed positive correlations between DUPrS rank and BVMT-R scores (<90th percentile of DUPrS rank) and CFT scores (within the 20th-70th percentile range of DUPrS rank). Among the 506 participants, 20.8% (95% CI, 17.4%-24.5%) converted to psychosis within 3 years. Cox proportional hazards regression analysis identified lower educational attainment (hazard ratio [HR], 0.912; 95% CI, 0.834-0.998), pronounced negative symptoms (HR, 1.044; 95% CI, 1.005-1.084), and impaired performance on the Neuropsychological Assessment Battery: Mazes (HR, 0.961; 95% CI, 0.924-0.999) and BVMT-R (HR, 0.949; 95% CI, 0.916-0.984) tests as factors associated with conversion.

**Conclusions and Relevance:**

The finding of this cohort study suggest the intricate interplay between DUPrS, cognitive performance, and conversion risk in individuals at CHR for psychosis. The findings emphasize the importance of considering both DUPrS and cognitive functioning in assessing the trajectory of these individuals.

## Key Points

**Question:**

Is the duration of untreated prodromal symptoms (DUPrS) associated with cognitive performance and risk of conversion to psychosis in individuals at clinical high risk (CHR) for psychosis?

**Findings:**

In this cohort study of 506 individuals at CHR for psychosis with 4 years’ follow-up, the DUPrS was associated with cognitive performance and the risk of conversion to psychosis. Shorter DUPrS was associated with poorer cognitive outcomes, particularly in tests such as the Brief Visuospatial Memory Test–Revised and Category Fluency.

**Meaning:**

The findings of this study suggest the importance of considering both the DUPrS and cognitive functioning in the evaluation of individuals at CHR for psychosis.

## Teaser

This cohort study examines the duration of untreated prodromal symptoms and cognitive function in individuals at clinical high risk for psychosis.

## Introduction

The association between the duration of untreated psychosis (DUP) and schizophrenia has been extensively examined,^1,2,3^ with a consistent finding that longer DUP is associated with more severe adverse outcomes,^4^ including cognitive impairments.^5,6^ However, recent attention has shifted toward understanding the pre–first-episode psychosis phase, termed clinical high risk (CHR) for psychosis.^7,8^ Individuals at CHR for psychosis exhibit attenuated positive symptoms that have not yet reached the severity of a full-blown psychotic episode, maintaining a degree of insight into their condition.^9,10^ Within this context, the period from the emergence of mild symptoms to seeking professional help is defined as the duration of untreated prodromal symptoms (DUPrS), a crucial yet understudied interval in the progression to psychosis.^11,12,13^ While previous research has explored the connection between DUP and cognitive impairments,^14,15^ the issue remains whether the association between DUPrS and cognitive function aligns with the established DUP-cognition association.

In the domain of individuals at CHR for psychosis, emerging evidence suggests the presence of cognitive impairments that lie between those observed in first-episode psychosis and those in healthy individuals.^16,17,18^ Furthermore, within the CHR population, those who eventually convert to full-blown psychosis exhibit more pronounced cognitive deficits than those who do not convert.^19,20^ These findings underscore the importance of investigating cognitive changes during this crucial period from attenuated symptoms to seeking professional assistance. This interval, termed the DUPrS, represents a pivotal yet underexplored phase in the pathway to psychosis. Understanding how DUPrS relates to cognitive functioning holds particular importance, as it sheds light on the early-stage cognitive alterations in individuals at CHR for psychosis. The intricate interplay between DUPrS and cognitive function in this ultra-early phase remains a research gap. This knowledge void is particularly salient given the potential implications for clinical intervention and conversion outcomes.

Given the existing knowledge gaps surrounding the association between DUPrS and cognitive functioning in individuals at CHR for psychosis, the present study aims to address this void. Our hypothesis posits that the length of DUPrS might be associated with how cognitive impairments manifest in individuals at CHR for psychosis. Specifically, we propose that individuals with longer DUPrS may exhibit distinct cognitive profiles compared with those with shorter DUPrS, potentially indicating variations in the severity and nature of cognitive deficits. Furthermore, we hypothesize that these cognitive differences could play a pivotal role in shaping the trajectory of individuals at CHR for psychosis toward conversion to psychosis. To test these hypotheses, we investigated DUPrS, cognitive performance, and conversion outcomes. By leveraging a comprehensive data set and using sophisticated statistical analyses, this study endeavored to uncover novel insights into the interplay between DUPrS and cognitive functioning, offering a more refined understanding of the complex dynamics underlying the conversion to psychosis.

## Methods

### Project and Procedure

This cohort study obtained data from an ongoing longitudinal investigation, the Shanghai at Risk for Psychosis–extended (SHARP-extended) program^21,22,23^ conducted from January 10, 2016, to December 29, 2021. The study participants, diagnosed with CHR for psychosis, were enrolled in an integrated clinical risk assessment and early psychosis intervention initiative at the Shanghai Mental Health Center. As China’s largest outpatient mental health clinic, Shanghai Mental Health Center offers comprehensive medication management and psychotherapy services. Individuals with a history of substance abuse or dependence were excluded from the study. Individuals who had received treatment with psychotropic medications at baseline also were excluded from the study. Ethical approval was secured from the Shanghai Mental Health Center Research Ethics Committee, and written informed consent, including parental consent for participants younger than 18 years, was diligently obtained. Participants did not receive financial compensation. This study followed the Strengthening the Reporting of Observational Studies in Epidemiology (STROBE) reporting guideline.

A team of 4 research assistants coordinated the follow-up procedures. Telephone conversations were conducted with individuals at CHR for psychosis every 6 months to monitor their medical status diligently. Participants were assured they could readily contact the research assistants for any queries. Upon reaching the 3-year milestone, participants were cordially invited to face-to-face interviews. The ascertainment of clinical outcomes primarily hinged on the insights garnered from these face-to-face interviews, spanning 3 years. Furthermore, clinical outcome assessments were enriched by telephone interviews involving participants and their caregivers and corroborated through cross-referencing with clinician reports and medical records. The information provided by the participants was considered primary, and in certain cases, particularly when these individuals were hospitalized, caregivers were contacted for additional information. If the information provided by caregivers was limited or unclear, relevant hospital or outpatient treatment records were requested for reference. The research team then collectively evaluated and discussed the individual’s outcome during clinical meetings, considering all available information before reaching a consensus.

### Participants

This study represents a subset of the larger primary investigation encompassing 1000 participants. Specifically, our focus was on individuals who had completed initial baseline neuropsychological assessments and underwent a 3-year clinical follow-up (N = 506). The sample size was not predetermined through formal calculations but rather based on the available cohort meeting the specified criteria within the given timeframe of the ongoing longitudinal investigation. While the sample size was not statistically determined in advance, its composition aligns with the criteria of interest and the scope of the study, reflecting the available population meeting the research objectives within the specified timeframe. Inclusion criteria involved individuals aged 14 to 45 years who fulfilled diagnostic criteria for 1 of 3 psychosis risk syndromes: (1) attenuated positive symptom syndrome, (2) brief intermittent psychotic syndrome, or (3) genetic risk and deterioration syndrome. These diagnoses were established using the Structured Interview for Prodromal Syndromes (SIPS) interview.^24^ Exclusion criteria encompassed a current or lifetime occurrence of a psychotic episode, symptoms that could be attributed more fittingly to nonpsychotic disorders or substance abuse, previous use of psychotropic medication regardless of dosage, ongoing or historical use of psychoactive substances (eg, methamphetamine), presence of neurologic or endocrine disorders, lack of proficiency in Mandarin, and an inability to comprehend or provide informed consent.

### Measurements and Variables

To identify individuals at CHR for psychosis, we conducted comprehensive face-to-face interviews using the SIPS. The SIPS comprises 19 items that assess positive symptoms (scales P1-P5), negative symptoms (scales N1-N6), disorganized symptoms (scales D1-D4), and general symptoms (scales G1-G4). Each item is rated on a 1 to 6 scale, where 6 indicates severe and psychotic, and items 3 to 5 indicate a prodromal range symptom. Criteria for the attenuated positive symptom syndrome are met by at least 1 positive symptom rated 3 to 5, occurring at least once a week, on average, in the last month, and either new within the past year or rated at least 1 point higher than 1 year earlier. The genetic risk and deterioration syndrome is indicated by a functional deterioration (30% decrease in global assessment of functioning score compared with 12 months earlier) in the context of either schizotypal personality disorder or at least 1 first-degree relative with psychosis. In previous investigations,^11,25^ our team introduced a Chinese version of the SIPS, showing robust interrater reliability (intraclass correlation coefficient: *r* = 0.96; *P* < .01; SIPS total score) and validity (26.4% of participants transitioned to psychosis within the subsequent 2 years) in the Chinese context. Four psychiatrists (T.Z., H.C., Y.W., and L.X.) with more than 5 years of clinical experience conducted the SIPS assessments in this study. Before commencement of the research, these evaluators underwent systematic training for SIPS interviews. The training involved assessing their proficiency through an individual scoring assessment after independently observing 2 training videos. Interrater reliability was assessed using κ values, showing agreement rates between 0.81 and 0.95. The interrater reliability for the SIPS positive symptoms ranged from 0.86 (P5) to 0.98 (P4) among the 4 raters. Additionally, the Cronbach α value for all SIPS items was 0.71.

The primary outcome of this study was the conversion to psychosis. This determination was made using predefined criteria involving psychotic symptoms as outlined by the SIPS framework. Specifically, the criterion for conversion encompassed the occurrence of a positive symptom with a severity rating of 6 on the 6-level scale, indicating severe and psychotic symptoms. This symptom needed to be distressing, disorganized, or persistent for at least 1 hour per day on average, occurring over a minimum of 4 days per week, totaling at least 16 hours. Retrospective evaluation of DUPrS encompassed the interval between the emergence of the initial attenuated psychotic positive symptom (reaching at least a moderate level, corresponding to a score of 3 or higher on the SIPS) as identified through the SIPS interview and the initiation of professional aid at mental health services.^11^

Neurocognitive functioning was evaluated using the Chinese version of the Measurement and Treatment Research to Improve Cognition in Schizophrenia Consensus Cognitive Battery (MCCB).^26^ The MCCB assessment adhered to the standardized guidelines outlined in the test manual. Consistent with the original MCCB, the study incorporated the following 8 subtests: (1) Trail Making Test Part A: participants connect numbered circles in ascending order as quickly as possible; (2) Symbol Coding in the Brief Assessment of Cognition in Schizophrenia (BACS) (BACS symbol coding): participants match symbols to numbers using a key; (3) Category Fluency Test: participants generate words belonging to a specific category within a set time; (4) Continuous Performance Test–Identical Pairs version: assesses sustained attention and vigilance; (5) Spatial Span in the Wechsler Memory Scale–Third Edition test: participants repeat sequences of spatial locations; (6) Hopkins Verbal Learning Test–Revised (HVLT-R): measures verbal learning and memory; (7) Brief Visuospatial Memory Test–Revised (BVMT-R): assesses visuospatial memory; and (8) Neuropsychological Assessment Battery: Mazes: participants complete mazes evaluating executive function. Higher scores represent better performance. Except for the Trail Making Test Part A, higher scores across these tests indicate better cognitive performance. The interrater reliability of the MCCB, based on ratings from the 4 trained assessors, ranged from 0.82 to 0.95.

To address potential sources of bias, we took steps to ensure the reliability and validity of data collection. The research team underwent extensive training for the application of the SIPS, promoting consistency and minimizing interrater variability. Additionally, rigorous measures were implemented during neurocognitive assessments using the Chinese version of the MCCB in the development and validation of which our research team actively participated. Recognizing the potential for recall bias during the assessment of DUPrS, we implemented strategies to minimize this bias. Participants were encouraged to provide specific times of symptom onset, and these reported times were cross-verified with their cohabiting relatives or companions, enhancing the reliability of DUPrS assessments and reducing potential recall bias.

### Statistical Analysis

Data were analyzed from August 25, 2021, to May 10, 2023. The statistical analysis was conducted using SPSS Statistics for Windows, version 20.0 (IBM Corp), with 2-sided, unpaired testing and a significance threshold of *P* < .05. Descriptive statistics provided means (SD) for quantitative variables and frequencies (percentage) for qualitative variables. Based on DUPrS percentiles (33rd percentile corresponding to 3 months, 66th percentile to 9 months), participants were categorized into short (≤3 months) (n = 166), median (4-9 months) (n = 181), and long (≥10 months) (n = 159) DUPrS groups. Nonparametric methods, such as the Kruskal-Wallis test for comparisons, were used due to nonnormal variable distributions. Neurocognitive *z* scores were created based on the total sample of 506 participants. This transformation was undertaken to facilitate better comparability among different variables in the graphic representations. While *z* scores were used for graphic comparisons, the Cox proportional hazards and quantile regression analyses used the original raw scores to ensure accuracy in statistical assessments. The correlation analysis is based on linear regression. The *F* values represent nonzero slope tests from linear regression. Quantile regression, adjusted for age, sex, years of education, and positive, negative, disorganized, and general symptoms, examined the cognitive performances across DUPrS percentiles between 0.1 and 0.9, with a step size of 0.1. Quantile regression plots depicted regression parameters along with 95% CIs. Quantile regression offers benefits over standard linear models, enabling the exploration of distribution points beyond the mean, and it is robust to data distribution assumptions, outlier sensitivity, and transformations, ensuring reliability across diverse scenarios.^27,28^ Finally, /yb5vg4rf regression was performed to identify baseline factors associated with conversion by the 3-year follow-up.

Although both quantile and Cox proportional hazards are regression analyses, in this study, they serve distinct purposes. Quantile regression was used to explore the association between DUPrS and cognitive function, where the outcome variable was cognitive function. Cox regression was used to examine the association between various variables and the time to psychosis onset, with the outcome variable being the occurrence of psychosis. Despite their shared regression framework, the methods of quantile regression and Cox regression differ substantially. Quantile regression focuses on estimating conditional quantiles of the dependent variable and is robust to outliers, providing insights into how the association between variables changes across different points of the distribution. In contrast, Cox regression, often used for survival analysis, assesses the hazard or risk of an event occurring over time, making it suitable for analyzing time-to-event outcomes such as the onset of psychosis. These methodologic distinctions align with the specific aims of each analysis in this study.

## Results

In total, 506 individuals at CHR for psychosis (271 [53.6%] women, 235 [46.4%] men) with a mean (SD) age of 19.2 (5.424) years and a median age of 19 (IQR, 16-21) years were included. The study sample was predominantly of Han ethnicity, with only 2 individuals of Uyghur ethnicity and 1 individual of Yi ethnicity. The proportion of genetic risk and deterioration syndrome (n = 35) and brief intermittent psychotic syndrome (n = 31) cases was relatively small compared with the predominant attenuated positive symptom syndrome cases (n = 476), minimizing their contribution to the overall results. The transition rates for the 3 subgroups were as follows: 22.6% for brief intermittent psychotic syndrome (7 of 31), 21.2% for attenuated positive symptom syndrome (101 of 476), and 37.1% for genetic risk and deterioration syndrome (13 of 35), with no statistically significant differences (*χ^2^* = 4.769; *P* = .09). The mean (SD) educational level, recorded as the number of years of education received by participants starting from primary school, was 10.8 (2.966) years.

 The median DUPrS was 6 (IQR, 3-11) months and the mean (SD) was 7.8 (6.857) months. Several significant differences were observed in the comparison among the short, median, and long DUPrS groups (Table 1). The short DUPrS group had a significantly lower score in negative symptoms than the median and long groups (Kruskal-Wallis χ**^2^** = 7.395; *P* = .03). The BVMT-R (Kruskal-Wallis χ^2^ = 8.801; *P* = .01) and Category Fluency Test (Kruskal-Wallis χ^2^ = 6.670; *P* = .04) test scores were significantly lower, indicating poorer performance in the short and median groups than in the long group.

**Table 1.** Demographic, Clinical, and Cognitive Characteristics

Variable	Total^a^ (N = 506)		DUPrS group						Comparison^b^	
			Short^c^ (n = 166)		Median^d^ (n = 181)		Long^e^ (n = 159)			
	Mean (SD)	Median (IQR)	Mean (SD)	Median (IQR)	Mean (SD)	Median (IQR)	Mean (SD)	Median (IQR)	χ^2^^f^	*P* value
Age, y	19.2 (5.4)	17 (15-21)	19.7 (5.9)	17 (16-23)	18.8 (5.2)	17 (15-20)	19.1 (5.2)	18 (15-21)	1.681	.43
Education, y	10.8 (3.0)	10 (7-12)	10.7 (3.0)	10 (9-12)	10.7 (2.9)	10 (9-12)	11.0 (3.1)	10 (9-13)	1.049	.60
Positive symptoms	9.7 (3.6)	10 (7-12)	10.1 (3.8)	10 (7-13)	9.6 (3.3)	10 (8-12)	9.5 (3.6)	9 (7-12)	2.584	.20
Negative symptoms	12.3 (5.9)	12 (8-17)	11.5 (5.9)	11 (7-16)	13.2 (5.9)	13 (9-18)	12.0 (5.9)	12 (7-16)	7.395	.02
Disorganization symptoms	6.1 (3.1)	6 (3-8)	6.2 (3.4)	6 (3-8)	5.9 (3.0)	6 (3-8)	6.1 (3.0)	6 (4-8)	0.079	.96
General symptoms	9.1 (3.0)	9 (7-11)	9.1 (3.2)	9 (7-12)	9.1 (2.7)	10 (8-11)	8.9 (3.1)	9 (7-11)	0.346	.84
Test scores^g^										
Trail Making Test Part A	33.4 (14.2)	31 (25-38)	34.3 (12.8)	32 (25-39)	33.3 (13.4)	31 (24-39)	32.7 (16.4)	30 (24-37)	3.919	.14
BACS symbol coding	56.7 (10.9)	57 (50-64)	56.5 (10.2)	57 (51-63)	55.8 (10.6)	56 (49-63)	58.0 (11.7)	59 (50-65)	3.581	.17
HVLT-R	23.6 (5.2)	24 (20-27)	22.7 (5.2)	23 (19-27)	23.9 (5.3)	24 (20-28)	24.1 (4.9)	24 (21-28)	6.266	.046
WMS-3 spatial span	15.5 (3.2)	16 (14-18)	15.3 (3.1)	15 (13-18)	15.6 (3.4)	16 (13-18)	15.5 (3.2)	15 (14-18)	1.598	.46
NAB mazes	16.8 (6.3)	17 (12-22)	16.5 (6.4)	18 (12-22)	16.7 (6.3)	17 (12-22)	17.2 (6.1)	18 (13-22)	0.962	.63
BVMT-R	25.7 (6.5)	27 (21-31)	24.4 (7.0)	26 (20-30)	26.1 (6.4)	27 (21-31)	26.7 (5.8)	28 (23-31)	8.801	.01
Category Fluency	19.5 (5.5)	19 (16-23)	19.3 (5.6)	19 (16-23)	18.9 (5.3)	19 (15-22)	20.4 (5.5)	20 (17-23)	6.670	.04
CPT-IP	2.4 (0.8)	2.5 (1.9-3.0)	2.3 (0.8)	2.4 (1.8-2.9)	2.4 (0.9)	2.5 (1.9-3.0)	2.4 (0.7)	2.4 (2.0-3.0)	2.227	.32

Abbreviations: BACS, Brief Assessment of Cognition in Schizophrenia symbol coding; BVMT-R, Brief Visuospatial Memory Test–Revised; CPT-IP, Continuous Performance Test–Identical Pairs; DUPrS, duration of untreated prodromal symptoms; HVLT-R, Hopkins Verbal Learning Test–Revised; NAB, Neuropsychological Assessment Battery: Mazes; WMS-3, Wechsler Memory Scale–Third Edition spatial span.

^a^ A total of 235 were male (46.4%) and 271 female (53.6%).

^b^ For comparison across gender, χ^2^ = 5.609; *P* = .06.

^c^ A total of 66 were male (39.8%) and 100 female (60.2%).

^d^ A total of 85 were male (47.0%) and 96 female (53.0%).

^e^ A total of 84 were male (52.8%) and 75 female (47.2%).

^f^ *χ^2^* for the Kruskal-Wallis test and κ test.

^g^ Except for the Trail Making Test Part A, higher scores across these tests indicate better cognitive performance.

**Figure 1.**
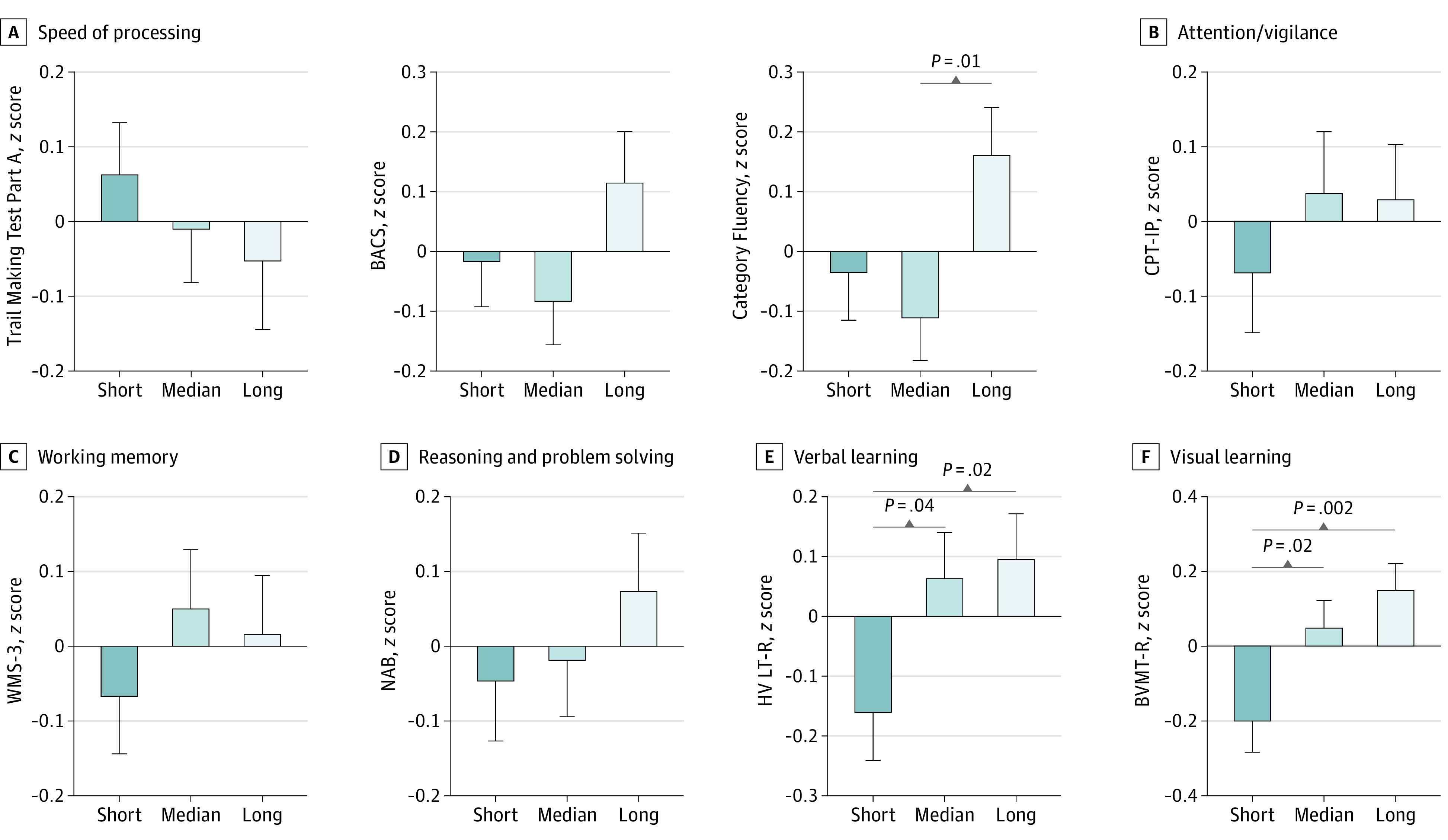
Distribution of Cognitive Variables Among Individuals at Clinical High Risk for Psychosis With Short (≤3 Months), Median (4-9 Months), and Long (≥10 Months) Duration of Untreated Prodromal Symptoms Mean *z* scores of performance tests, with error bars indicating SD. BACS indicates Brief Assessment of Cognition in Schizophrenia symbol coding; BVMT-R, Brief Visuospatial Memory Test–Revised; CPT-IP, Continuous Performance Test–Identical Pairs; HVLT-R, Hopkins Verbal Learning Test–Revised; NAB, Neuropsychological Assessment Battery: Mazes; and WMS-3, Wechsler Memory Scale–Third Edition spatial span.

Overall, the short and median DUPrS groups showed poorer performance in cognitive tests than the long DUPrS group (Figure 1). Regarding HVLT-R, individuals in the short DUPrS group scored lower, indicating poorer performance, than those in the median and long groups (Figure 1). Similarly, individuals in the short DUPrS group showed lower scores, indicating poorer performance, in BVMT-R than those in the median and long groups. In the Category Fluency Test, individuals in the median DUPrS group showed lower scores, indicating poorer performance, than those in the long DUPrS group (Figure 1).

### Correlations

**Figure 2.**
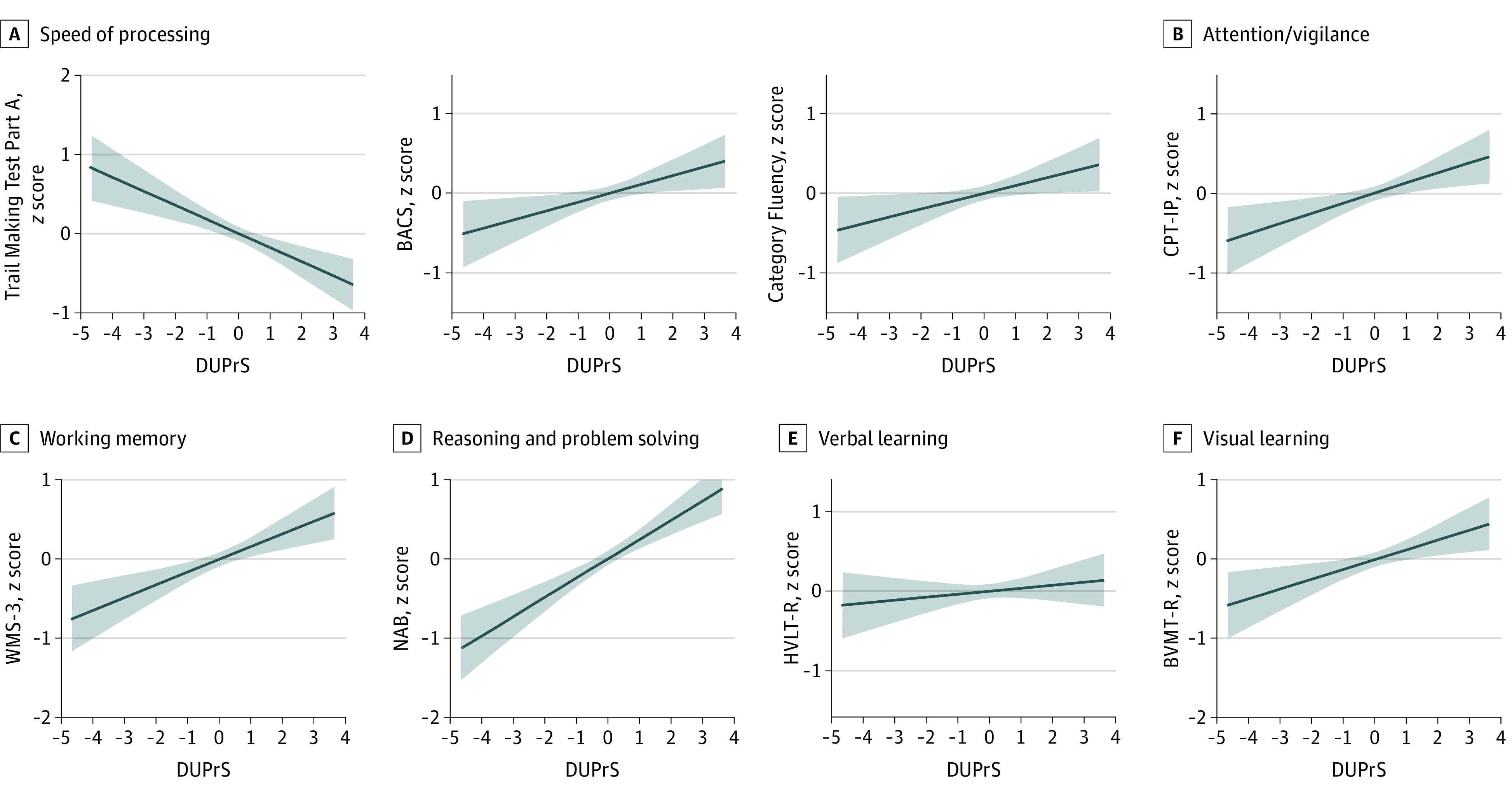
Correlations Between Duration of Untreated Prodromal Symptoms (DUPrS) and Cognitive Characteristics, Adjusted by Age, Gender, and Years of Education *F* values displayed on the graphs were derived from the nonzero slope tests in linear regression. Shaded areas indicate SD. BACS indicates Brief Assessment of Cognition in Schizophrenia symbol coding; BVMT-R, Brief Visuospatial Memory Test–Revised; CPT-IP, Continuous Performance Test–Identical Pairs; HVLT-R, Hopkins Verbal Learning Test–Revised; NAB, Neuropsychological Assessment Battery: Mazes; and WMS-3, Wechsler Memory Scale–Third Edition spatial span.

Adjusted for age, sex, and educational level, DUPrS were negatively and significantly correlated with the score of the Trail Making Test Part A. The DUPrS were positively and significantly correlated with almost all other subtests except the Spatial Span in the Wechsler Memory Scale–Third Edition spatial span test (Figure 2).

### Quantile Regression

**Figure 3.**
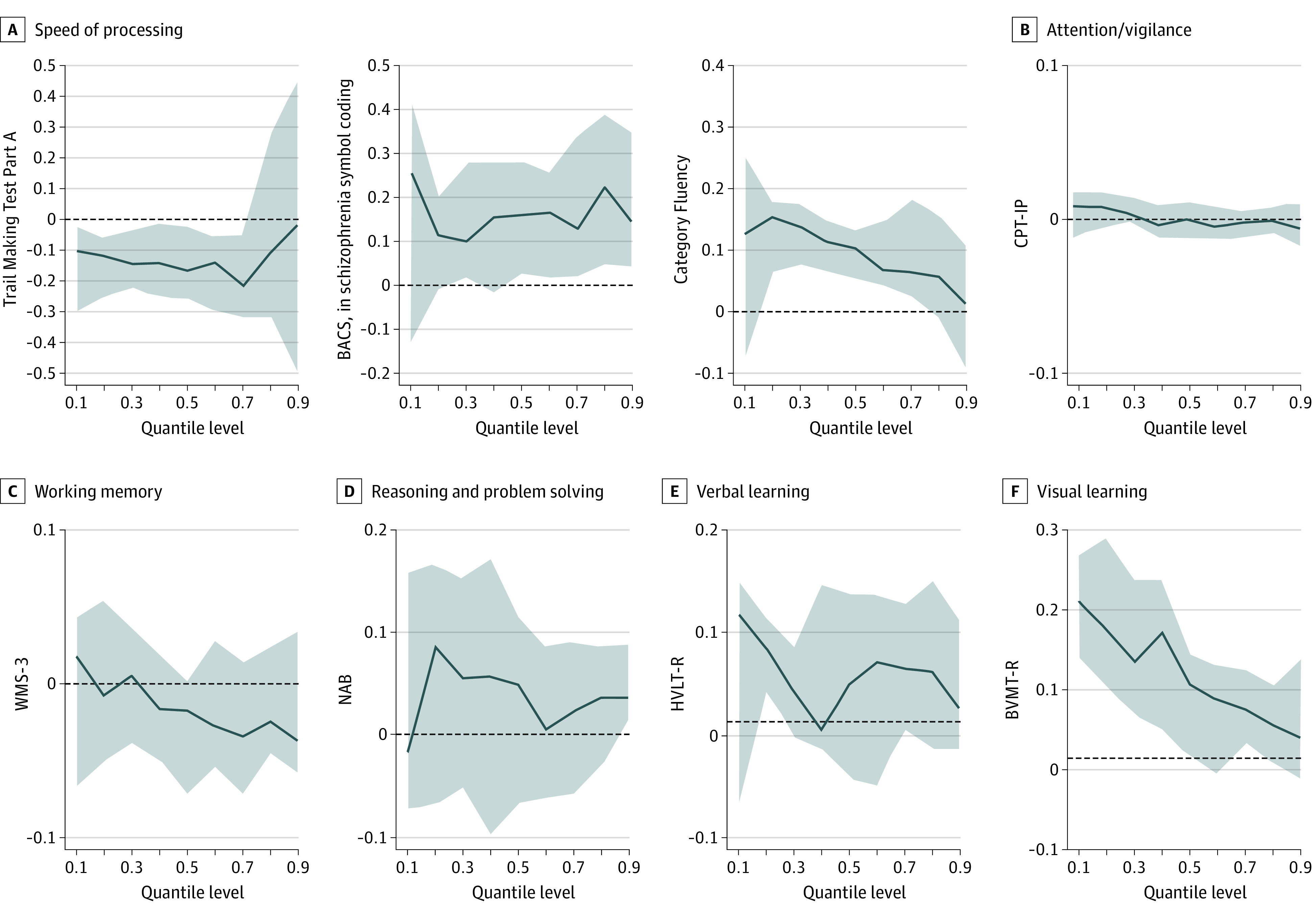
Quantile Regression Plots for Cognitive Performances of Duration of Untreated Prodromal Symptoms (DUPrS) Percentile Ranks P10, P20, P30, P40, P50, P60, P70, P80, and P90 Quantile regression models with cognitive variables including Trail Making Test Part A; Brief Assessment of Cognition in Schizophrenia symbol coding (BACS); Hopkins Verbal Learning Test–Revised (HVLT-R); Wechsler Memory Scale–Third Edition (WMS-3) spatial span; Neuropsychological Assessment Battery (NAB) mazes; Brief Visuospatial Memory Test–Revised (BVMT-R); Category Fluency Test; Continuous Performance Test–Identical Pairs (CPT-IP) as the dependent variables and the DUPrS as the independent variable for the quantiles between 0.1 to 0.9, with increments of 0.1, adjusted for age, sex, education, and positive, negative, disorganized, and general symptoms. Coefficient estimates were calculated with the independent variables in terms of percentiles, and they represent the association with the dependent variable for each 1-decile increase in the independent variable.

Figure 3 presents the estimated quantile regression parameters for various cognitive variables across all quantiles (0.1-0.9) of DUPrS ranks. Negative associations were noted between DUPrS and Trail Making Test Part A scores below 0.7 for all quantiles. Conversely, positive associations were observed between DUPrS rank and BACS symbol coding scores above quantile 0.5, BVMT-R below quantile 0.9, and Category Fluency Test scores within the 0.2- to 0.7-quantile range (Figure 3).

### Cox Proportional Hazards Regression

Among the 506 participants, 105 (20.8%; 95% CI, 17.4%-24.5%) converted to psychosis in the following 3 years. Cox regression was used to assess demographic, clinical, and cognitive factors and the risk of conversion to psychosis. This model identified lower educational attainment (hazard ratio [HR], 0.912; 95% CI, 0.834-0.998; *P* = .045), pronounced negative symptoms (HR, 1.044; 95% CI, 1.005-1.084; *P* = .03), and impaired performance on Neuropsychological Assessment Battery: Mazes (HR, 0.961; 95% CI, 0.924-0.999; *P* = .04) and BVMT-R (HR, 0.949; 95% CI, 0.916-0.984; *P* = .004) as significant conversion factors (Table 2). Within the short DUPrS group (38 of 166 individuals progressed to psychosis), female gender and subpar results in BVMT-R and Category Fluency tests emerged as factors associated with conversion risk. The model in the median DUPrS group (41 of 181 participants progressed to psychosis) highlighted that only severe positive symptoms were associated with conversion risk. In contrast, the long DUPrS group (28 of 159 individuals progressed to psychosis) displayed a distinct pattern, with poor BVMT-R performance associated with conversion risk.

**Table 2.** Cox Regression for Symptomatic and Cognitive Variables Predicting Conversion to Psychosis

Variables	Analysis^a^			
	β (SE)	HR (95% CI)	χ^2^	*P* value
Overall model				
Age	0.032 (0.023)	1.033 (0.988 to 1.080)	1.992	.16
Sex	0.439 (0.226)	1.552 (0.997 to 2.416)	3.786	.05
Education	−0.092 (0.046)	0.912 (0.834 to 0.998)	4.033	.045
Positive symptom	0.060 (0.031)	1.062 (0.999 to 1.130)	3.684	.06
Negative symptom	0.043 (0.019)	1.044 (1.005 to 1.084)	4.899	.03
Disorganized symptom	0.026 (0.037)	1.026 (0.955 to 1.102)	0.484	.49
General symptom	−0.022 (0.037)	0.978 (0.910 to 1.051)	0.359	.55
Trail Making Test Part A	−0.003 (0.008)	0.997 (0.982 to 1.013)	0.120	.73
BACS symbol coding	−0.002 (0.012)	0.998 (0.976 to 1.021)	0.021	.89
HVLT-R	0.024 (0.023)	1.024 (0.978 to 1.071)	1.041	.31
WMS-3 spatial span	0.052 (0.037)	1.054 (0.980 to 1.133)	1.996	.16
NAB mazes	−0.040 (0.020)	0.961 (0.924 to 0.999)	4.144	.04
BVMT-R	−0.052 (0.018)	0.949 (0.916 to 0.984)	8.135	.004
Category Fluency Test	0.026 (0.020)	1.027 (0.986 to 1.068)	1.650	.20
CPT-IP	0.041 (0.155)	1.042 (0.768 to 1.412)	0.069	.79
DUPrS	−0.001 (0.016)	0.991 (0.959 to 1.023)	0.345	.56
Short DUPrS				
Sex	1.081 (0.424)	2.949 (1.285 to 6.769)	6.506	.01
BVMT-R	−0.070 (0.032)	0.932 (0.876 to 0.992)	4.876	.03
Category Fluency Test	0.098 (0.038)	1.103 (1.024 to 1.187)	6.729	.009
Median DUPrS				
Positive symptoms	0.119 (0.056)	1.126 (1.010 to 1.257)	4.540	.03
Long DUPrS				
BVMT-R	−0.102 (0.040)	0.903 (0.834 to 0.977)	6.423	.01

Abbreviations: BACS, Brief Assessment of Cognition in Schizophrenia symbol coding; BVMT-R, Brief Visuospatial Memory Test–Revised; CPT-IP, Continuous Performance Test–Identical Pairs; DUPrS, duration of untreated prodromal symptoms; HR, hazard ratio; HVLT-R, Hopkins Verbal Learning Test–Revised; NAB, Neuropsychological Assessment Battery: Mazes; WMS-3, Wechsler Memory Scale–Third Edition spatial span.

^a^ Subgroup analyses included the same set of variables as the overall Cox model, and only significant variables are presented.

## Discussion

This study observed distinct patterns in the associations between DUPrS and cognitive performance, as well as the risk of conversion to psychosis. Short and median DUPrS groups demonstrated poorer cognitive performance than long DUPrS groups. Shorter DUPrS was associated with lower scores in HVLT-R and BVMT-R, while median DUPrS showed lower scores in Category Fluency Tests. Quantile regression analysis further underscored these associations across different quantiles of DUPrS. Cox regression identified lower educational attainment, pronounced negative symptoms, impaired performance on Neuropsychological Assessment Battery mazes, and BVMT-R were associated with increased conversion risk to psychosis. These associations were particularly pronounced within the short DUPrS group, where female gender and cognitive performance in BVMT-R and Category Fluency Tests emerged as significant factors. The median DUPrS group demonstrated a distinct factor—severe positive symptoms. In contrast, the long DUPrS group displayed a unique pattern, with poor BVMT-R performance associated with conversion risk. These findings collectively highlight the intricate interplay between DUPrS, cognitive functioning, and conversion risk, offering valuable insights into the multifaceted dynamics of psychosis development in individuals at CHR for psychosis.

The short DUPrS group exhibited poorer cognitive performance than the median and long DUPrS groups. Specifically, we observed associations between DUPrS and 2 cognitive domains: verbal (HVLT-R) and visual (BVMT-R) learning. Participants in the short DUPrS group displayed significantly lower scores than those in both the median and long DUPrS groups. This finding suggests that individuals at CHR for psychosis with a rapid symptom onset may experience compromised verbal learning and visuospatial memory abilities,^20,29,30^ potentially linked to the urgency of symptom manifestation leading to cognitive impairment. The intriguing disparity in Category Fluency Test scores between individuals in the median and long DUPrS groups invites a more nuanced exploration of the underlying dynamics. This finding raises the issue of why this outcome is observed specifically in the median DUPrS group rather than the short DUPrS group. One plausible explanation could be rooted in the subtleties of symptom manifestation and the cognitive processes involved in semantic fluency. This intermediate phase might represent a delicate balance between the rapid and intense symptom onset associated with the short DUPrS group and the more gradual progression seen in the long DUPrS group.

In contrast to previous findings suggesting an association between DUP and poorer cognitive function in patients with first-episode psychosis,^31,32,33^ our study uncovers a perplexing connection between longer DUPrS and improved cognitive function. This counterintuitive pattern can be attributed to the fundamental distinction between DUPrS and traditional DUP. While DUP primarily reflects the temporal delay from the onset of psychosis to treatment, focusing on the consequences of treatment delay,^34,35^ DUPrS captures the interval from attenuated symptom emergence to help-seeking. This emphasis on the process of individuals at CHR for psychosis recognizing psychotic issues at an ultra-early stage is closely related to the urgency of symptom manifestation.^12^ This distinction highlights the unexpected negative correlation between DUPrS and cognitive impairments. Specifically, as DUPrS lengthens, the manifestation of attenuated symptoms becomes more covert. This subtler presentation might delay patients’ recognition of the need for intervention, resulting in a potentially smaller degree of cognitive impairment. To elaborate further, consider an individual with a short DUPrS marked by abrupt and intense symptoms, which prompts early help-seeking. In contrast, another individual with a longer DUPrS reflects gradual and subtle symptom progression that might not immediately trigger recognition of the need for assistance. However, we acknowledge the lack of statistically significant baseline differences among DUPrS subgroups in terms of positive symptoms. In-depth analysis of positive symptom subitems (P1-5) revealed that, while total scores did not exhibit significant differences, the short DUPrS group demonstrated a higher proportion of scores of 5 or 6 in these items of P2-suspiciousness and P4-perceptual abnormalities compared with the median and long DUPrS groups. This nuanced examination aligns with our hypothesis that a shorter DUPrS may be indicative of more intense symptoms, potentially contributing to cognitive impairment. This intricate interplay between symptom dynamics, awareness, and cognitive functions underscores the relevance of DUPrS as a pivotal variable shaping the cognitive landscape in individuals at CHR for psychosis.

In considering the observed differences in DUPrS groups, it is essential to recognize the potential influence of various factors beyond the progression of symptom onset. While our main interpretation emphasizes the rapid and intense symptom onset associated with the short DUPrS group and the more gradual progression seen in the long DUPrS group, alternative explanations should be considered. Factors such as individuals’ sensitivity to symptoms, comfort with seeking care, access to care, support systems, and stigma could significantly impact the DUPrS. Individuals in the short DUPrS group might have sought care more rapidly due to heightened awareness, accessibility to mental health services, or a supportive environment. Additionally, the motivation for seeking help related to cognitive symptoms could have played a role, potentially leading to shorter DUPrS. Our study did not explicitly measure these factors, and we acknowledge the need for further research to explore the complex interplay between symptom experiences, help-seeking behaviors, and the DUPrS. Moreover, in considering the potential cognitive resilience in the long DUPrS group, we acknowledge the possibility that these individuals may have developed adaptive cognitive strategies over time, coping with the chronicity of their symptoms. Although our study did not directly measure these adaptive processes, recognizing the potential influence of cognitive adaptation provides a more comprehensive understanding of the intricate relationships between symptom experiences, help-seeking behaviors, and cognitive trajectories during the prodromal phase.

Furthermore, our findings encourage a deeper exploration of specific cognitive assessments. The consistent positive correlation observed between DUPrS and cognitive functions related to processing speed (eg, Trail Making Test Part A, BACS symbol coding, and Category Fluency) below the 0.7 quantiles suggests that individuals with a rapid onset of symptoms may exhibit a distinct cognitive resilience. This prompts us to delve into the potential differences in the cognitive development trajectories between the short DUPrS (indicative of acute onset) and long DUPrS (reflecting gradual progression) groups. Particularly intriguing is the observation that the processing speed is directly proportional to DUPrS in the short DUPrS group. This might stem from swift symptom onset in this group, triggering a heightened cognitive response and enhancing their processing speed. This phenomenon could be attributed to an adaptive mechanism where the urgency of symptom manifestation prompts an immediate cognitive mobilization, potentially explaining the observed correlation.^36^ However, such a correlation might not hold in the long DUPrS group due to the subtler and gradual nature of symptom progression. Overall, these nuanced relationships emphasize the importance of recognizing the intricate interplay between DUPrS, cognitive function, and symptom dynamics in individuals at CHR for psychosis, shedding light on potential variations in cognitive trajectories based on the temporal characteristics of symptom onset.

Using Cox proportional hazards regression enabled us to comprehensively evaluate how demographic, clinical, and cognitive aspects may influence the risk of transitioning to psychosis. Our study identified several noteworthy factors within distinct DUPrS groups, shedding light on the complex interplay of variables in the trajectory toward psychosis: lower educational attainment, pronounced negative symptoms, and impaired cognitive function in reasoning, problem-solving, and visual learning across all DUPrS groups. The short DUPrS group displayed a unique constellation of risk factors for conversion. Female gender and impaired cognitive function in the speed of processing and visual learning emerged as significant factors associated with conversion risk. This intriguing pattern suggests that gender-related factors and specific cognitive domains might synergistically contribute to heightened vulnerability within this subgroup. In the median DUPrS group, severe positive symptoms emerged as a significant factor of conversion risk. This finding emphasizes the pivotal role of positive symptoms in the mid-range DUPrS group. Conversely, the long DUPrS group exhibited a distinct association pattern. Poor performance on the BVMT-R was identified as a factor associated with conversion risk, indicative of the potential cognitive deficits of visual learning associated with a lengthier interval DUPrS. This observation points to the relevance of cognitive domains related to visual memory and spatial processing^19,20^ in the conversion process within this particular subgroup.

### Limitations

This study is subject to several limitations. First, the sample was drawn from a single site, enhancing homogeneity but potentially limiting the generalizability of the findings. Second, the absence of accounting for individual variations in baseline cognitive abilities influenced by IQ^37,38^ could impact result interpretation. Future studies should include IQ assessments to comprehensively understand cognitive differences. Third, in consideration of the relatively low *R*^2^ values observed in our correlational analysis, it is important to approach the interpretation of these correlations with caution. The modest explained variance highlights the complexity of the association between DUPrS and cognitive measures, and caution should be exercised when generalizing these findings. Fourth, the naturalistic observation of the SHARP-extended cohort and various psychotropic medications, including antipsychotics and antidepressants, could introduce confounding factors.^39,40,41^ Fifth, our analysis did not consider socioeconomic status, income, and perceived stigma, which are known to influence DUP. Furthermore, the inclusion of multiple variables in our analyses increases the risk of type I errors. Additionally, the potential selection bias introduced by including only participants with both baseline and 3-year follow-up data may limit the generalizability of our findings. It is essential to acknowledge that despite our efforts to analyze and compare baseline characteristics between participants who completed the 3-year follow-up assessments and those who did not, there may still be some residual selection bias. While the conducted analyses did not reveal statistically significant differences between the 2 groups, the possibility of unmeasured confounders or factors influencing the completion of follow-up assessments cannot be entirely ruled out. Sixth, our study did not explicitly measure the specific characteristics of symptom onset, such as whether it was more insidious or sudden. This limitation leaves room for uncertainty regarding the nature of symptom onset in the longer DUPrS group and the potential influence of other factors contributing to the delay in seeking care. Future research endeavors should aim to explore symptom onset trajectories in more depth to enhance our understanding of the underlying mechanisms influencing the observed differences between DUPrS groups.

## Conclusions

The findings of our cohort study illuminate the intricate association between DUPrS, cognitive performance, and the risk of conversion to psychosis in individuals at CHR for psychosis. The investigation revealed that shorter DUPrS was associated with poorer cognitive outcomes, with specific cognitive domains affected differently across different DUPrS categories. The results underscore the need for comprehensive assessments that consider DUPrS and cognitive functioning when evaluating the trajectory of individuals at CHR for psychosis. Future research could delve into the underlying mechanisms that contribute to these observed associations and explore potential interventions to optimize cognitive outcomes and mitigate conversion risk in this vulnerable population.

## Notes

Supplement.
Data Sharing Statement
