# Supplementary material for: Duration of Untreated Prodromal Psychosis and Cognitive Impairments
Source: JAMA Netw Open. 2024 Jan 26;7(1):e2353426. doi: 10.1001/jamanetworkopen.2023.53426 (PMC10818213; doi:10.1001/jamanetworkopen.2023.53426)
Supplement: Supplement. — Data Sharing Statement [file jamanetwopen-e2353426-s001.pdf]

Data Sharing Statement

Zhang. Duration of Untreated Prodromal Psychosis and Cognitive Impairments. *JAMA Netw Open*. Published January 26, 2024. doi:10.1001/jamanetworkopen.2023.53426

Data
Data available: Yes
Data types: Deidentified participant data
How to access data: Data will be available based on the reasonable request from Dr. TianHong Zhang, email: zhang_tianhong@126.com
When available: With publication

Supporting Documents
Document types: None

Additional Information
Who can access the data: researchers whose proposed use of the data has been approved
Types of analyses: for a specified purpose
Mechanisms of data availability: with a signed data access agreement
